# The evolution of clot strength in critically-ill COVID-19 patients: a prospective observational thromboelastography study

**DOI:** 10.1186/s12959-021-00331-5

**Published:** 2021-11-06

**Authors:** Colette Neethling, Gregory Calligaro, Malcolm Miller, Jessica J. S. Opie

**Affiliations:** 1 Department of Anaesthesia & Perioperative Medicine, University of Cape Town, Groote Schuur Hospital, Anzio Drive, Observatory, Cape Town, 7925 South Africa; 2 Division of Pulmonology, Department of Medicine, University of Cape Town, Groote Schuur Hospital, Cape Town, South Africa; 3 Division of Haematology, Department of Pathology, University of Cape Town & National Health Laboratory Service, Groote Schuur Hospital, Cape Town, South Africa

**Keywords:** Corona virus disease 2019 (COVID-19), Thromboelastography (TEG), Acute respiratory distress syndrome (ARDS), Hypercoagulability, COVID-19 coagulopathy, Critically-ill

## Abstract

**Background:**

Few studies detail the evolution of COVID-19 associated coagulopathy. We performed serial thromboelastography (TEG) and laboratory coagulation studies in 40 critically-ill, mechanically ventilated COVID-19 patients over a 14-day period and analysed differences between 30-day survivors and non-survivors.

**Methods:**

Single-center prospective, observational study including 40 patients with severe COVID-19 pneumonia admitted to the intensive care unit (ICU) for mechanical ventilation. TEG analysis was performed on days 1, 7 and 14 of ICU admission and laboratory coagulation studies were performed on days 1 and 14. Coagulation variables were evaluated for change over the 14-day observation period. Differences between survivors and non-survivors at 30-days were analysed and compared.

**Results:**

On admission, TEG maximum amplitude (MA) with heparinase correction was above the upper limit of the reference range in 32 (80%) patients while 33 (82.5%) presented with absent clot lysis at 30 min. The functional fibrinogen MA was also elevated above the upper limit of the reference range in 37 (92.5%) patients. All patients had elevated D-dimer and fibrinogen levels, mildly prolonged prothrombin times (PT), normal platelet counts and normal activated partial thromboplastin times (aPTT). The heparinase MA decreased significantly with time and normalised after 14 days (*p* = < 0.001) while the increased fibrin contribution to clot strength persisted with time (*p* = 0.113). No significant differences in TEG analysis were noted between 30-day survivors and non-survivors at all time points. No patients developed disseminated intravascular coagulopathy (DIC) after 14-days, however thrombosis and bleeding were each reported in 3 (7.5%) patients.

**Conclusion:**

Critically-ill patients with COVID-19 present in a hypercoagulable state characterised by an increased clot strength. This state normalises after 14 days despite a persistently increased fibrin contribution to clot strength. We were unable to demonstrate any significant differences in TEG parameters between 30-day survivors and non-survivors at all time points.

**Supplementary Information:**

The online version contains supplementary material available at 10.1186/s12959-021-00331-5.

## Background

In December 2019 a pneumonia-like illness emerged in Wuhan, Hubei Province in Central China. This was caused by the novel coronavirus Severe Acute Respiratory Syndrome Coronavirus 2 (SARS-CoV-2) [1]. Coronavirus disease 2019 (COVID-19) has since spread rapidly across the globe, affecting most nations worldwide. At the time of writing, global confirmed cases exceed 238 million with over 4.8million recorded deaths [2]. COVID-19 is associated with increased risk of venous thromboembolism (VTE) [3–7] and a hypercoagulable state characterised by increased clot strength and hypofibrinolysis on viscoelastic tests (VET) [8–11]. The four key mechanisms regarded to be responsible for this prothrombotic state include widespread endothelial damage, activation of coagulation and platelets, and suppression of fibrinolysis [12].

Thromboelastography (TEG) is a point of care VET which demonstrates clotting and fibrinolytic activity of whole blood in vitro. It was first used to identify bleeding abnormalities and guide blood transfusion strategies [13, 14]. More recently TEG has been validated to identify hypercoagulable states and evaluate risk for thromboembolic events [14, 15]. Most studies have evaluated VET in critically-ill COVID-19 patients at a single time point [8–11]. A minority have evaluated patients at various time points with all demonstrating a persistent hypercoagulable state [16–22], however differences between survivors and non-survivors are unclear. It is known that COVID-19 associated coagulopathy plays an integral role in morbidity and mortality with more pronounced derangements in the severely ill [23, 24]. We therefore hypothesise that hypercoagulability lessens in time, especially in those who survive. This prospective, observational study therefore aimed to describe the evolution of coagulopathy over a 14-day period and to identify any differences in coagulation parameters between 30-day survivors and non-survivors.

## Methods

### Study design and subjects

This was a single center, prospective, observational study which included 40 consecutive patients admitted to the intensive care unit (ICU), between July 19, and August 13, 2020 at Groote Schuur Hospital, a tertiary academic center in Cape Town, South Africa. Ethics approval was obtained from the Human Research and Ethics Committee of the Faculty of Health Sciences of the University of Cape Town (HREC 400/2020). Delayed informed consent was obtained for survivors and proxy consent for non-survivors. All patients admitted to ICU during the study period with severe COVID-19 pneumonia, as defined by the World Health Organisation, were eligible for enrolment [25]. Study patients were intubated and mechanically ventilated and had laboratory confirmed SARS-CoV-2 by reverse transcription-polymerase chain reaction. Patients were excluded if they were younger than 18 years of age, pregnant, known with active cancer or an underlying bleeding disorder.

### Standard of care

As per local institutional guidelines, all patients with severe COVID-19 pneumonia with ratios of arterial oxygen partial pressure to fractional inspired oxygen (Pa0_2_/Fi0_2_) < 100 were first placed on high flow nasal oxygen (HFNO) therapy in a high care ward owing to severe constraints on ICU beds. Patients on HFNO were initiated on glucocorticoid therapy (6 mg dexamethasone) once daily for 10 days and therapeutic anticoagulation with either enoxaparin 1 mg/kg twice daily or unfractionated heparin (UFH) infusion, depending on the clinical scenario, and in the absence of contraindications [26]. Enoxaparin was monitored using anti-Factor Xa levels, targeting a therapeutic range of 0.6–1.0 IU/ml. UFH was monitored targeting an activated clotting time (ACT) of 180-220 s. If patients deteriorated clinically on HFNO therapy, they were intubated and transferred to ICU for mechanical ventilation. Glucocorticoid and anticoagulation therapy continued in the ICU. Laboratory coagulation studies included D-dimer and fibrinogen (ACL TOP 500 CTS, HemosIL reagents), platelet count (Sysmex XN-10 with Fluorocell WNR, Lysercell WNR, Cellpak DCL reagents), prothrombin time (PT) (ACL TOP 500 CTS, HemosIL RecombiPlasTin 2G reagents) and activated partial thromboplastin time (aPTT) (ACL TOP 500 CTS, HemosIL SynthASil reagents). Imaging for VTE was performed at the discretion of the treating physician when clinically indicated.

### Data and sample collection

Laboratory coagulation studies were performed on day 1 and 14 of ICU admission using samples collected from an arterial line. TEG analysis was performed on day 1, 7 and 14 of ICU admission using blood from 3.2% sodium citrate Vacutainer® tubes, as per manufacturers recommendations. TEG analysis was performed within 30 min of sample collection and not specifically timed to heparin dosing. The same operator conducted the TEG analysis in all cases to prevent inter-operator variability. Patient information collected included: age, sex, body mass index (BMI),comorbidities, anticoagulation use/dosage, glucocorticoid use/dosage, date of symptom onset and ICU admission. Disease severity scores included the Pa0_2_/Fi0_2_ ratio to classify severity of acute respiratory distress syndrome (ARDS) [27], the sequential organ failure assessment (SOFA) score to quantify organ involvement [28], and the International Society on Thrombosis and Haemostasis (ISTH) disseminated intravascular coagulation (DIC) score to determine rate of DIC [29]. Outcomes measured included survival or death at 30 days and bleeding or thrombotic complications. Bleeding events were included if they met the criteria for major bleeding according to the Control of Anticoagulation Subcommittee of the ISTH [30]. Thrombotic complications included those confirmed on radiological imaging, where requested by the treating physician.

### Thromboelastography

TEG analysis was performed using the TEG®6 s analyser (Haemonetics®, Braintree, MA).

Results from two TEG reagents were recorded. The first TEG was performed with citrated kaolin in heparinase to neutralise the effect of heparin. The second, TEG functional fibrinogen, contains tissue factor and a glycoprotein IIb/IIIa platelet receptor inhibitor to isolate the contribution of fibrinogen to clot strength [31]. TEG parameters recorded included the R-time, K-time, α-angle, heparinase maximum amplitude (HMA), functional fibrinogen maximum amplitude (FFMA) and clot-lysis at 30 min (LY30). Hypercoagulability was defined as an HMA above the upper limit of the reference range (> 68 mm) [32]. Patient values were compared to the reference ranges provided by the TEG 6 s operating manual.

### Statistical analysis

Statistical analysis was performed using STATA/SE. Data for descriptive analysis was reported as mean (standard deviation [SD]), median (interquartile range [IQR]) or number (%) as appropriate. Differences between 30-day survivors and non-survivors at all time points were analysed using the Student t-test, Mann-Whitney U test, χ^2^ test or Fisher’s exact test as appropriate. Data were tested for differences at various time points using a Friedman, Wilcoxon, ANOVA and Paired t-test as appropriate. A *p*-value of less than 0.05 was deemed statistically significant.

## Results

Forty patients were included in the study. All patients presented with severe ARDS requiring mechanical ventilation with median admission SOFA scores of 4 [4–6] and PaO_2_/FiO_2_ ratios of 95 (64–124). One patient developed acute kidney injury (AKI) requiring renal replacement therapy and one required extracorporeal membrane oxygenation (ECMO). The mean age was 55 years (±8) and 26 patients (65%) were male. Thirty-four patients (85%) were overweight or obese with a BMI > 25 kg/m^2^ [33], 28 (70%) had hypertension and 27 (67.5%) diabetes. The median time from onset of symptoms to ICU admission was 11.5 days [8–14]. Thirty-nine patients (97.5%) received therapeutic anticoagulation; 37 (92.5%) of these received enoxaparin and 2 (5%) UFH due to acute renal failure and ECMO therapy respectively. One patient (2.5%) received prophylactic dose heparin, due to a perceived increased bleeding risk by the treating physician. Clinically significant bleeding events occurred in three patients (7.5%) whilst receiving therapeutic anticoagulation. These included pulmonary haemorrhage requiring massive blood transfusion, intracranial haemorrhage leading to brain stem death and epistaxis requiring cauterization and blood transfusion. Anticoagulation was discontinued in all these patients due to uncontrolled bleeding complications. Confirmed large vessel thrombosis occurred in 3 (7.5%) patients despite therapeutic anticoagulation. These included one case each of pulmonary embolus, cardiac thrombus and lower limb deep venous thrombosis. All patients with a bleeding or thrombotic event did not survive and no patients experienced simultaneous thrombosis and haemorrhage. Detailed TEG analysis of the bleeding and thrombotic group can be found in the supplementary figures.

**Fig. 1:**
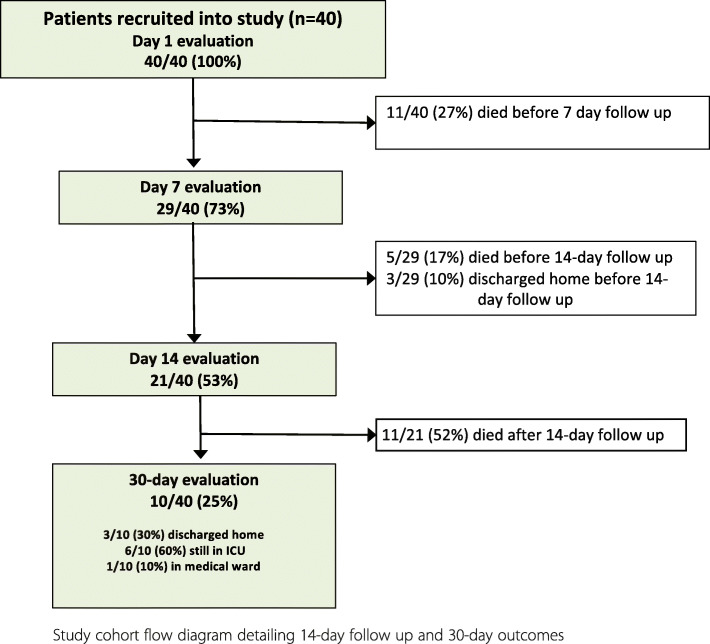
Study cohort flow diagram

At 30-day follow up, 6 patients (15%) remained in ICU, one (2.5%) patient had been discharged to the medical ward, 6 (15%) had been discharged home and 27 (67.5%) had demised with a median time from ICU admission to death of 11 days [4–19]. Figure 1 details the 14-day follow up and 30-day outcomes of study patients.

Table 1 displays baseline characteristics, laboratory coagulation and TEG values of the study patients. Admission laboratory coagulation studies demonstrated a mildly prolonged PT, elevated D-dimer and fibrinogen level, normal platelet counts and normal aPTT. The median DIC score on admission was 3 [2, 3] with only one patient meeting the ISTH criteria for DIC [29]. Admission TEG analysis confirmed a hypercoagulable state, with an HMA above the upper limit of the reference range in 32 (80%) patients and absent clot lysis at 30-min in 33 (82.5%). FFMA was also above the upper limit of reference range in 37 (92.5%) patients. The only significant differences noted between the 30-day survivor and non-survivor groups on admission were BMI (*p* = 0.02) and urea level (p = 0.02).

**Table 1:** Baseline clinical characteristics, laboratory coagulation and thromboelastography parameters on admission to the intensive care unit

Variable on admission	Reference ranges	Total (***N*** = 40)	30-day Survivor (***N*** = 13)	30-day Non-survivor (***N*** = 27)	***P*** value
Age, years (SD)		55 (±8)	52 (±9)	56 (±8)	0.09
**Sex**					0.08
Female n (%)		14 (35)	7 (54)	7 (26)	
Male n (%)		26 (65)	6 (46)	20 (74)	
**Comorbidities**					
Hypertension n (%)		28 (70)	9 (69)	19 (70)	0.94
Diabetes n (%)		27 (68)	8 (62)	19 (70)	0.58
**BMI** (**kg/m**^**2**^**)**					0.02
Normal weight (18–24.9) n (%)		6 (15)	0 (0)	6 (22)	
Overweight (25–29.9) n (%)		13 (33)	2 (15)	11 (41)	
Obese (> 30) n (%)		21 (52)	11 (85)	10 (37)	
**Disease Severity Scores**					
SOFA score (IQR)		4 (4–6)	4 (4–4)	4 (4–6)	0.55
Pa0_2_/Fi0_2_ ratio (IQR)	200 > − ≤ 300 Mild ARDS 100 > − ≤ 200 Moderate ARDS ≤ 100 Severe ARDS	95(64–124)	95 (60–103)	96 (66–130)	0.61
DIC score (IQR)	< 5 not suggestive of DIC ≥ 5 is suggestive of DIC	3 (2–3)	3 (2–3)	3 (2–4)	0.55
**Laboratory Values**					
Platelet count, × 10^9^/L (IQR)	171–388	354 (267–448)	352 (263–466)	356 (270–438)	0.86
Urea, mmol/L (IQR)	2.1–7.1	9.5 (7.3–11.6)	7.7 (5.8–8.5)	10.2 (9.1–13.0)	0.02
Creatinine, μmol/L (IQR)	64–104	72 (61.5–117.0)	67 (64–81)	85 (61–136)	0.32
Prothrombin time, s (IQR)^a^	11–12.5	13.7 (13.0–15.3)	13.3 (12.8–13.5)	14.2 (13.2–15.7)	1.00
Activated partial thromboplastin time, s (IQR)	25.1–36.5	30.1 (28.6–32.8)	29.9 (28.9–32.6)	30.2 (28.4–32.8)	1.00
D-Dimer, mg/L (IQR)	< 0.25	1.2 (0.6–3.1)	0.9 (0.6–2.5)	1.4 (0.6–4.0)	1.00
Fibrinogen, g/L (IQR)	2–4	5 (5–5)^b^	5 (5–5)^b^	5 (5–5)^b^	0.59
**TEG Heparinase**					
R-time, min (IQR)	4.3–8.3	7.7 (5.8–9.8)	7.7 (7.5–8.2)	7.8 (5.8–10.7)	0.87
K-time, min (IQR)	0.8–1.9	1.1 (0.9–1.7)	1.1 (0.9–1.4)	1.2 (0.9–1.8)	0.46
α-angle, degree (IQR)	64–77	75.4 (69.6–77.9)	75.0 (71.3–78.9)	75.9 (69.6–77.9)	0.87
Maximum amplitude, mm (IQR)	52–68	70.3 (68.5–72.1)	71.1 (69.5–72.6)	70.2 (68.5–71.7)	0.30
LY-30, % (IQR)	0–8	0 (0)	0 (0–0.1)	0 (0)	0.70
**TEG Functional Fibrinogen**					
Maximum amplitude, mm (IQR)	15–32	47.0 (41.2–52.6)	48.3 (41.4–50.4)	44.9 (40.2–52.6)	0.88

Table 1**.** Demographics, laboratory coagulation studies and thromboelastography (TEG) results for all study patients on admission to the intensive care unit (ICU). Differences between admission characteristics for overall 30-day survivors (*N* = 13) and non-survivors (*N* = 27) are shown with corresponding *p*-values. Data are reported as mean (±SD), median (IQR), n (%). Reference ranges were established by the local laboratory and TEG 6 s operating manual.

*BMI* Body mass index, *SOFA* Sequential organ failure assessment, *Pa0*_*2*_*/Fi0*_*2*_ Ratio of arterial oxygen partial pressure to fractional inspired oxygen, *DIC* Disseminated intravascular coagulopathy, *PT* Prothrombin time, *aPTT* Activated partial thromboplastin time

^a^PT laboratory control for the duration of the study was 11.7 s

^b^Value is above the upper measurable limit of fibrinogen in local laboratory

Of the 40 patients recruited, 11 died within 7 days of ICU admission and could not be followed up over the 14-day observation. Comparisons of the admission characteristics, laboratory coagulation and TEG values for the early death group (*N* = 11) and 30-day survivors (*N* = 13) are shown in Table 2.

**Table 2:** Baseline characteristics of the 30-day survivors and early death group (died before 7 days)

Variable on admission	Normal reference ranges	30-day Survivor (***N*** = 13)	Died before 7 days (***N*** = 11)	***P*** value
Age, years (SD)		52 (±9)	57 (±9)	0.171
Female, n (%)		7 (54)	1 (9)	0.046
**Comorbidities**				
Hypertension, n (%)		9 (69)	7 (64)	0.556
Diabetes, n (%)		8 (62)	8 (73)	0.444
Overweight, (BMI > 25 kg/m^2^) n (%)		2 (15)	6 (55)	0.055
Obese, (BMI > 30 kg/m^2^) n (%)		11 (85)	2 (18)	0.002
**Disease Severity Scores**				
SOFA score (IQR)		4 (4–4)	5 (4–7)	0.360
PaO_2_/FiO_2_ ratio (IQR)		95 (60–103)	101 (81–122)	0.450
DIC score (IQR)		3 (2–3)	3 (3–4)	0.113
**Laboratory Values**				
Platelet count, × 10^9^/L (IQR)	171–388	352 (263–466)	358 (255–438)	0.776
Urea, mmol/L (IQR)	2.1–7.1	7.7 (5.8–8.5)	9.3 (6.1–12.8)	0.296
Creatinine, μmol/L (IQR)	64–104	67 (64–81)	65 (56–136)	0.943
Prothrombin time, s (IQR)^a^	11–12.5	13.3 (12.8–13.5)	15.0 (13.6–15.8)	0.013
Activated partial thromboplastin time, s (IQR)	25.1–36.5	29.9 (28.9–32.6)	30.2 (28.7–33.3)	0.809
D-Dimer, mg/L (IQR)	< 0.25	0.9 (0.6–2.5)	3.0 (0.9–5.3)	0.096
Fibrinogen, g/L (IQR)	2–4	5 (5–5)^b^	5 (5–5)^b^	0.399
**TEG Heparinase**				
R time, min (IQR)	4.3–8.3	7.7 (7.5–8.2)	6.6 (5.4–9.7)	0.579
K time, min (IQR)	0.8–1.9	1.1 (0.9–1.4)	1.0 (0.8–2.0)	0.830
α-angle, degree (IQR)	64–77	75.0 (71.3–78.9)	77.3 (66.6–78.7)	0.679
Maximum amplitude, mm (IQR)	52–68	71.1 (69.5–72.6)	70.5 (69.1–71.7)	0.503
LY-30, % (IQR)	0–8	0 (0–0.1)	0 (0–0.6)	0.529
**TEG functional fibrinogen**				
Maximum amplitude, mm (IQR)	15–32	48.3 (41.4–50.4)	42.1 (39.4–49.1)	0.353

Table 2 Demographics, laboratory coagulation studies and thromboelastography (TEG) results for the overall 30-day survivors (*N* = 13) compared to the early death group (*N* = 11) with corresponding *p*-values provided. Data are reported as mean (±SD), median (IQR), n (%). Reference ranges were established by the local laboratory and TEG 6 s operating manual.

*BMI* body mass index, *SOFA* Sequential organ failure assessment, *Pa0*_*2*_*/Fi0*_*2*_ Ratio of arterial oxygen partial pressure to fractional inspired oxygen, *DIC* Disseminated intravascular coagulopathy, *PT* Prothrombin time, *aPTT* Activated partial thromboplastin time

^a^PT laboratory control for the duration of the study was 11.7 s

^b^Value is above the upper measurable limit of fibrinogen in local laboratory

**Fig. 2:**
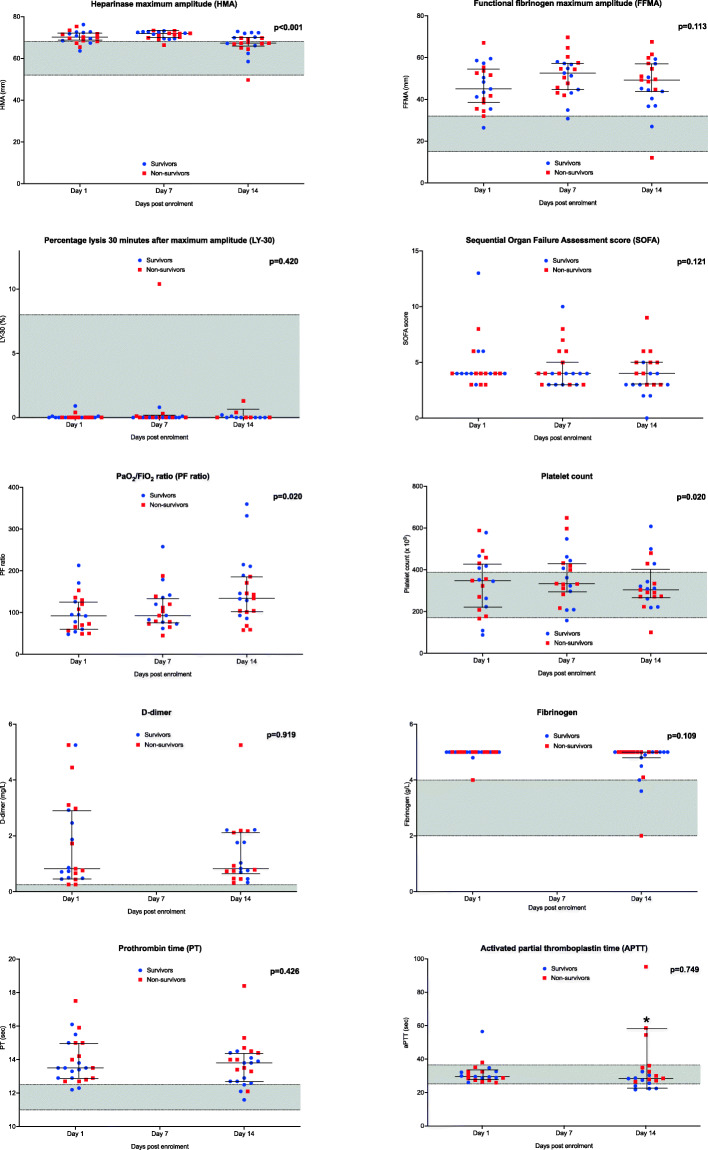
Evolution in laboratory coagulation tests, thromboelastography parameters and disease severity scores over 14-day period. Figure shows temporal changes in laboratory coagulation studies, thromboelastography (TEG) parameters and disease severity scores for 21 patients that survived to 14-day evaluation. 30-day survivors and non-survivors are represented as blue and red respectively. Coloured dots represent individual measurements. Grey band represents normal reference ranges as established by the local laboratory and the TEG 6 s operating manual. Asterix (*) indicates significant differences noted between survivors and non-survivors at specific time points. Data is presented as median (IQR), *p*-values shown in graphs represent changes over 14-day observation for all patients followed up. ULN = upper limit of normal, LLN = lower limit of normal

Due to the early deaths and discharges, only 21 of the 40 patients could be evaluated for evolution of coagulopathy over the 14-day observation. Figure 2 illustrates temporal changes in disease severity scores, laboratory coagulation and TEG values over the 14-day observation for these 21 patients. Elevated D-dimer, fibrinogen levels and mildly prolonged PT persisted with time. Platelet counts and aPTT remained within normal reference ranges. HMA decreased significantly over the 14-day period returning to the normal reference range (*p* = < 0.001) while the increased FFMA persisted (*p* = 0.113). The PaO_2_/FiO_2_ ratios improved significantly with time (*p* = 0.020). Table 3 details differences between 30-day survivors and non-survivors at all timepoints. No significant differences were noted except for aPTT and SOFA on day 14 (*p* = < 0.01, *p* = 0.01).

**Table 3:** Differences in coagulation tests, thromboelastography parameters and disease severity scores for 30-day survivors and non-survivors

Parameter	Reference ranges	Survivors (***N*** = 10)			Non-survivors (***N*** = 11)			***P***-value		
		*Day 1*	*Day 7*	*Day 14*	*Day 1*	*Day 7*	*Day 14*	*Day 1*	*Day 7*	*Day 14*
**D-dimer, mg/L (IQR)**	< 0.25	0.80 (0.5–2.46)		0.93 (0.7–1.77)	0.82 (0.44–3.1)		0.79 (0.47–2.17)	0.88		0.94
**Fibrinogen, g/L (IQR)**	2–4	5 (5–5)^b^		4.95 (4.5–5)	5 (5–5)^b^		5 (5–5)^b^	1.00		1.00
**Prothrombin time**^**a**^**, s (IQR)**	11–12.5	13.45 (12.9–13.8)		12.8 (12.5–13.9)	14 (12.8–15)		14 (13.4–14.7)	0.36		0.12
**Activated partial thromboplastin time, s (IQR)**	25.1–36.5	29.7 (28.9–32.6)		26.45 (22.4–28.6)	28.8 (27.2–32.7)		32.5 (27.8–54.4)	0.45		< 0.01
**Platelet count, X10**^**9**^**/L (IQR)**	171–388	348.5 (223–418)	342.5 (209–444)	316 (261–429)	348 (208–458)	334 (311–432)	291 (271–334)	0.76	0.50	0.39
**HMA, mm (IQR)**	52–68	71.3 (68.5–72.6)	72.3 (69.5–72.8)	70.05 (66.1–72.3)	70.1 (68.2–71.8)	71.3 (69.9–72)	67.3 (65.1–69.4)	0.62	0.46	0.19
**FFMA, mm (IQR)**	15–32	46.65 (41.2–57.3)	51.95 (43.1–56.7)	44.15 (36.9–50.6)	41.7 (35.5–53.7)	54.4 (45.6–60.4)	51.1 (48.5–59.9)	0.70	0.40	0.07
**SOFA score (IQR)**		4 (4–6)	4 (3–4)	3 (2–4)	4 (3–4)	4 (3–6)	5 (3–6)	0.37	0.29	0.01
**Pa0**_**2**_**/Fi0**_**2**_ **ratio (IQR)**		94 (60–126)	106.5 (77–142)	168 (129–215)	73 (57–130)	93 (73–120)	104 (68–146)	0.80	0.48	0.06

Table 3**:** Differences in laboratory coagulation studies, thromboelastography (TEG) parameters and disease severity scores for the 30-day survivors (*N* = 10) and non-survivors (*N* = 11) over the 14-day observation. Differences at all time points between 30-day survivors and non-survivors are shown with *p*-values provided. Data presented as median (IQR). Reference ranges as established by the local laboratory and TEG 6 s operating manual.

*HMA* Maximum amplitude with citrated kaolin and heparinase, *FFMA* Functional fibrinogen maximum amplitude, *SOFA* Sequential organ failure assessment, *Pa0*_*2*_*/Fi0*_*2*_ Ratio of arterial oxygen partial pressure to fractional inspired oxygen; *PT* Prothrombin time, *aPTT* Activated partial thromboplastin time

^a^PT laboratory control for the duration of the study was 11.7 s

^b^Value is above the upper measurable limit of fibrinogen in local laboratory

## Discussion

Our study describing the evolution of COVID-19 associated coagulopathy in a group of critically-ill, mechanically ventilated patients using TEG demonstrates four main findings. The first is a hypercoagulable state on ICU admission, with increased fibrin contribution to clot strength. The second is impaired fibrinolysis with 82.5% showing no clot lysis at 30-min on ICU admission. The third is that the hypercoagulable state evolved in time with overall clot strength returning to normal after 14-days. Lastly, no significant differences in TEG parameters were noted at all time points between 30-day survivors and non-survivors. Standard laboratory coagulation studies demonstrated markedly elevated D-dimer and fibrinogen levels on admission which persisted over the observation period. Although the PT was marginally prolonged throughout, the platelet count and aPTT always remained within the reference range over the 14-day observation period.

COVID-19 is associated with an increased rate of VTE in critically-ill patients despite adequate dose thromboprophylaxis, in keeping with an underlying hypercoagulable state [3–7]. Standard laboratory coagulation tests are limited in their ability to reveal the complexities of this hypercoagulable state. Platelet counts are generally within the normal reference range with PT and aPTT only mildly prolonged [34]. The D-dimer, a fibrin degradation product and marker of disease severity [23, 24], is elevated in severe cases indicating increased clot formation. Postmortem findings have demonstrated platelet-fibrin microthrombi in the lungs [35] regarding it to be the source of the raised D-dimer levels [36]. The D-dimer, however, is non-specific and can be raised in various other conditions. Fibrinogen, an acute phase reactant, is also elevated in hospitalised COVID-19 patients; however, the absolute value offers little information on fibrinogen function and interaction with platelets. These limitations have sparked heightened interest in VET such as ROTEM®, TEG®, ClotPro® and Quantra® which provide a comprehensive overview of coagulation in vitro from clot initiation, formation and lysis. Many studies have evaluated VET in critically-ill COVID-19 patients at one time point and have described a profound hypercoagulable state [8–11]. However serial VET would provide deeper insight into the complex interaction between inflammation and thrombosis over time.

There is little data on the evolution of coagulopathy using VET. Hulshof et al. [16] enrolled 36 critically-ill patients and followed them up over a 6-week period using ROTEM®. They reported a persistence in hypercoagulability and hypofibrinolysis over time. Cordier et al. [20] followed up 10 patients using the TEG 5000. On admission patients with COVID-19 had increased clot strength compared to the control group. These 10 patients were followed up and TEG was repeated at ICU discharge, revealing a further increase in clot strength. They concluded that the hypercoagulable state persists, even in patients with good outcomes. Correa et al. [21] evaluated 30 patients on day 0,1,3,7 and 14 of ICU admission using ROTEM and confirmed that increased clot strength persisted over time. These previous three studies show a persistence in hypercoagulability, which is contrary to our findings. Here we report a significant trend toward normal clot strength in those that survived to the 14-day evaluation. This could be for various reasons. In our cohort, all patients were initiated on dexamethasone prior to ICU admission. The RECOVERY collaborative group revealed a decrease in 28-day mortality in mechanically ventilated patients treated with dexamethasone [37]. The routine use of dexamethasone in all our patients may have played an integral role in abating the inflammatory response and contributing to the subsequent decrease in hypercoagulability seen after 14 days. The normalisation of clot strength in our group compared to the literature could also be attributed to the timing of our patients admission to ICU. In a resource rich setting, patients would be intubated and enrolled into VET studies at an earlier stage of the disease process. In our setting, due to severe resource constraints, patients with severe ARDS were first placed on HFNO, and thus were evaluated at a more advanced stage of disease. Although we did not demonstrate any significant changes in D-dimer and fibrinogen over time, the normalisation of clot strength on TEG could correspond with clinical recovery.

The anticoagulation strategy adopted locally at our institution could also impact our findings. All patients admitted to our ICU had been fully anticoagulated with enoxaparin or UFH. Apart from its anticoagulation properties, heparin has anti-inflammatory and potentially anti-viral properties too [38]. Some VET studies support this improvement in hypercoagulability after initiation of anticoagulation. Ranucci et al. [19] recruited 10 patients into their study, performing VET with the Quantra® analyser. They initiated aggressive anticoagulation with LMWH 6000-8000 IU BD and clopidogrel and reported an improvement in hypercoagulability after 14 days of increased anticoagulation. Bocci et al. [17] embarked on a similar study to evaluate the effect of full therapeutic anticoagulation on 40 critically-ill COVID-19 patients using TEG®6 s. They found that therapeutic anticoagulation had no impact on TEG parameters over 7 days. We also did not demonstrate significant differences between admission and day 7 clot strength; however, there was a significant difference on day 14. Similar findings may have been demonstrated had Bocci et al. continued their follow up to 14 days.

Despite the overall clot strength normalising in our study patients, we did find that the increased contribution of fibrin to clot strength persisted in time, confirming many previous reports [16, 17, 19, 21]. However, our findings contradict with those of Pavoni et al. [22] who reported a decreased contribution of fibrin to clot strength over time and normalisation on day 10. The main assays used to determine fibrin contribution to clot strength are FIBTEM (which uses tissue factor and cytochalasin D) and TEG functional fibrinogen (which uses tissue factor and abiximab) [39]. The FIBTEM assay has been shown to correlate with fibrinogen levels (r = 0.74) [16] and relies largely on fibrinogen for clot formation [16]. Despite this, overall clot strength correlates with platelet count and not fibrinogen levels [16]. We demonstrated similar findings as our HMA correlated with platelet count (r = 0.54, *p* < 0.001) and fibrinogen to FFMA (r = 0.41, *p* = 0.01). This indicates that although fibrinogen plays an important role in increased clot strength, other factors such as platelets are also important. Studies have revealed that SARS-CoV-2 induces platelet hyperreactivity with increased activation and aggregation [40]. Platelets have also shown greater adhesion and spread on fibrinogen and collagen in those with severe COVID-19 disease [40]. The TEG functional fibrinogen however has limitations as cytochalasin D has been proven to be far superior at inhibiting platelets than abiximab [39]. Also, in the presence of thrombocytosis, which is frequently seen in COVID-19 patients, abiximab is less successful at inhibiting platelets resulting in an over-estimation of fibrin contribution to clot strength [41]. Detailed platelet function analysis would prove more instructive. We were unable to demonstrate any significant differences in TEG parameters between 30-day survivors and non-survivors at all time points. This questions the usefulness of TEG in the clinical setting to risk stratify critically-ill COVID-19 patients and improve their care.

This novel serial VET study shows improvement in hypercoagulability in critically-ill COVID-19 patients as denoted by clot strength after 14-days and compares TEG parameters in 30-day survivors and non-survivors. The main study limitation is the relatively small sample size. Only 21 patients could be evaluated for VET changes over time which limits generalisability of the results. A further limitation is that our laboratory could only measure D-dimer and fibrinogen levels up to 5.25 mg/L and 5 g/L respectively. Therefore, the true fibrinogen and D-dimer values could not be measured and are likely to be underestimated. The expensive running cost of the TEG 6 s instrument further restricted the frequency with which patients could be evaluated. Ideally, we would have performed follow up TEG testing until the outcome of death or discharge was reached. Further study would include a larger patient cohort, with more frequent and prolonged follow up of TEG to clearly illustrate the trends identified in this study. Comprehensive evaluation of platelet function would also provide better insights into the relative contributions of platelets and fibrinogen in overall clot strength.

## Conclusion

Our study demonstrates that the increased clot strength seen in critically-ill COVID-19 patients normalises after 14-days, implying clinical recovery. The increased fibrin contribution to clot strength however appears to persist over time. We were unable to demonstrate any significant differences in TEG parameters between 30-day survivors and non-survivors. Further study is needed to assess the utility of TEG in identifying those at risk of poor outcomes.

## Supplementary Information

**Additional file 1: Figure 1.** Detailed thromboelastography tracings for the pulmonary haemorrhage patient. **Figure 2.** Detailed thromboelastography tracings for the epistaxis patient. **Figure 3.** Detailed thromboelastography tracings for the intracranial haemorrhage patient. **Figure 4.** Detailed thromboelastography tracing for the patient with a deep venous thrombosis. **Figure 5.** Detailed thromboelastography tracing of the patient with a cardiac thrombus. **Figure 6.** Detailed thromboelastography tracing for the pulmonary embolus patient.

## Data Availability

The datasets used and/or analysed during the current study are available from the corresponding author on reasonable request.

## Abbreviations

SARS-CoV-2: Severe acute respiratory syndrome coronavirus 2
TEG: Thromboelastography
R: Reaction
K: Kinetic
HMA: Heparinase maximum amplitude
FFMA: Functional fibrinogen maximum amplitude
MA: Maximum amplitude
α: Alpha
LY-30: Lysis of clot at 30 min
COVID-19: Coronavirus disease 2019
ICU: Intensive care unit
PT: Prothrombin time
aPTT: Activated partial thromboplastin time
IQR: Interquartile range
SD: Standard deviation
VTE: Venothromboembolism
VET: Viscoelastic test
ULN: Upper limit of normal
LLN: Lower limit of normal
