# Supplementary material for: The evolution of clot strength in critically-ill COVID-19 patients: a prospective observational thromboelastography study
Source: Thromb J. 2021 Nov 6;19:83. doi: 10.1186/s12959-021-00331-5 (PMC8572064; doi:10.1186/s12959-021-00331-5)
Supplement: Supplementary file 1 — Additional file 1: Figure 1. Detailed thromboelastography tracings for the pulmonary haemorrhage patient. Figure 2. Detailed thromboelastography tracings for the epistaxis patient. Figure 3. Detailed thromboelastography tracings for the intracranial haemorrhage patient. Figure 4. Detailed thromboelastography tracing for the patient with a deep venous thrombosis. Figure 5. Detailed thromboelastography tracing of the patient with a cardiac thrombus. Figure 6. Detailed thromboelastography tracing for the pulmonary embolus patient. [file 12959_2021_331_MOESM1_ESM.zip › DVT patient .pdf]

Figure 4: Detailed thromboelastography tracing for the patient with a deep venous thrombosis

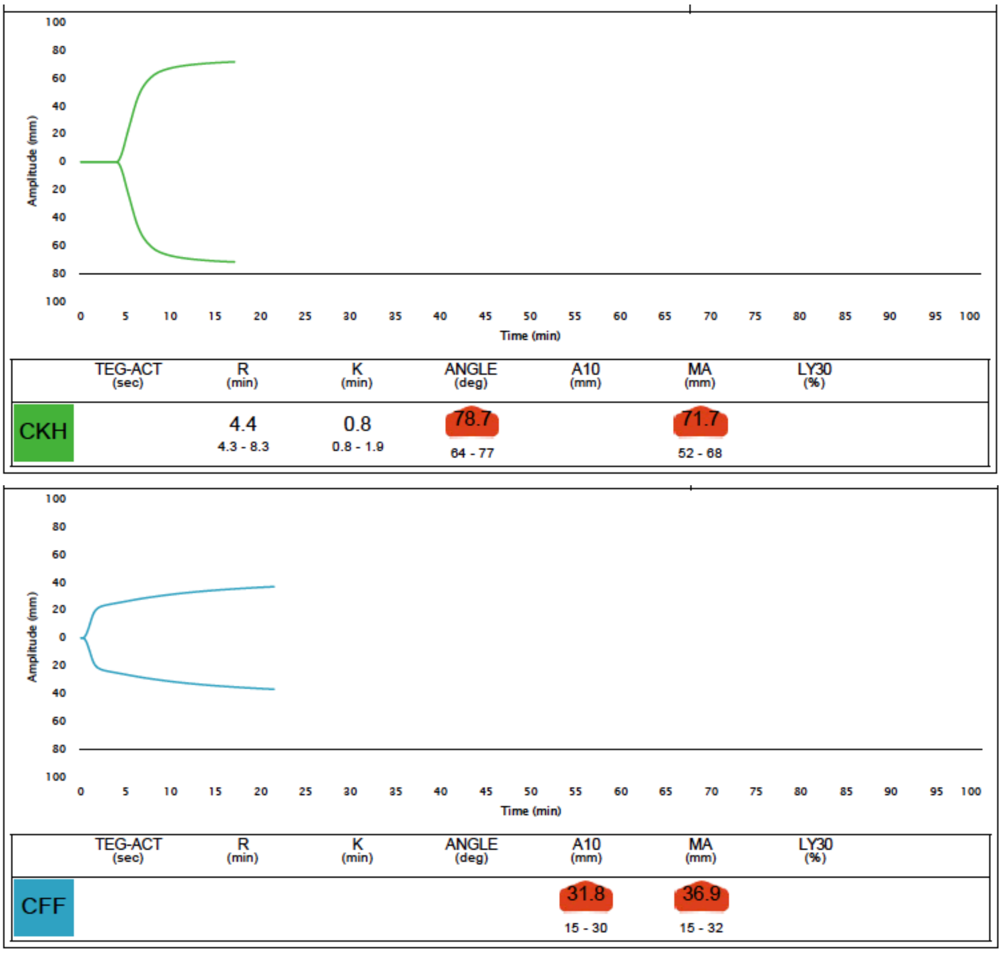

Day 1 TEG tracing

Figure 4: Detailed thromboelastography (TEG) tracings for the patient that developed a lower limb deep venous thrombosis on day 2 of admission. Therapeutic anticoagulation was continued. CKH=citrated kaolin in heparinise, CFF=citrated functional fibrinogen. Normal reference ranges as provided by the TEG 6s operating manual.
