# Supplementary material for: The evolution of clot strength in critically-ill COVID-19 patients: a prospective observational thromboelastography study
Source: Thromb J. 2021 Nov 6;19:83. doi: 10.1186/s12959-021-00331-5 (PMC8572064; doi:10.1186/s12959-021-00331-5)
Supplement: Supplementary file 1 — Additional file 1: Figure 1. Detailed thromboelastography tracings for the pulmonary haemorrhage patient. Figure 2. Detailed thromboelastography tracings for the epistaxis patient. Figure 3. Detailed thromboelastography tracings for the intracranial haemorrhage patient. Figure 4. Detailed thromboelastography tracing for the patient with a deep venous thrombosis. Figure 5. Detailed thromboelastography tracing of the patient with a cardiac thrombus. Figure 6. Detailed thromboelastography tracing for the pulmonary embolus patient. [file 12959_2021_331_MOESM1_ESM.zip › Pulmonary haemorrhage .pdf]

Figure 1: Detailed thromboelastography tracings for the pulmonary haemorrhage patient

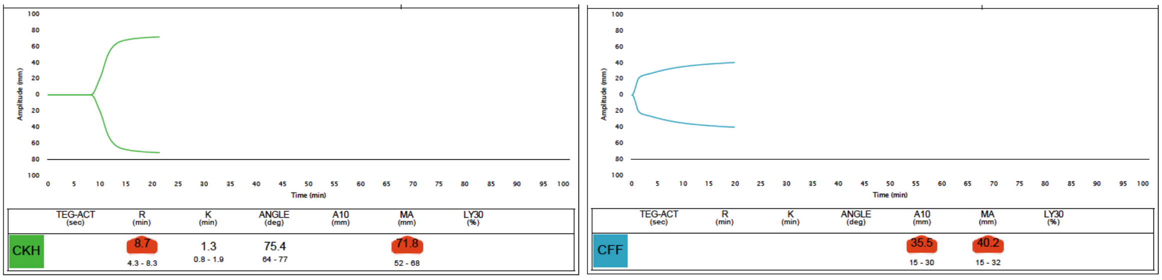

Day 1 TEG tracing

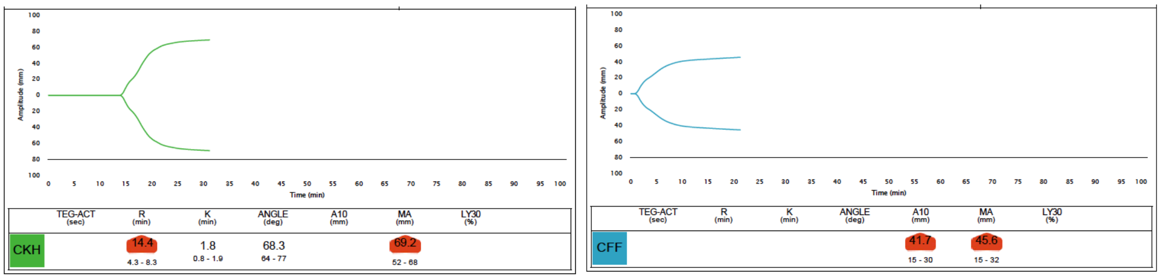

Day 7 TEG tracing

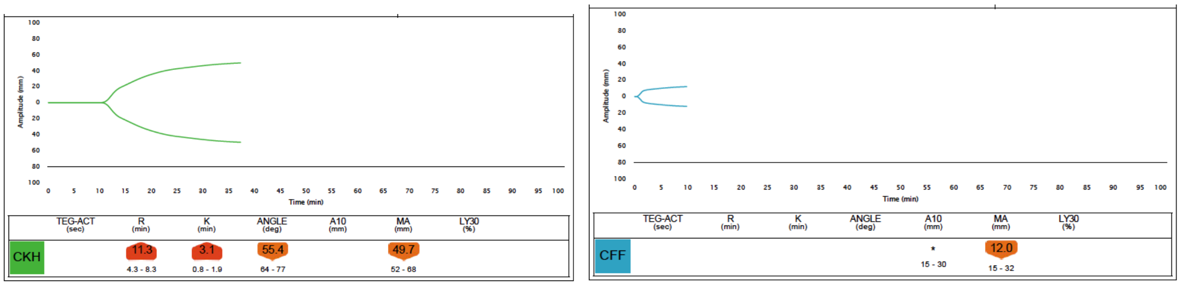

Day 14 TEG tracing

Figure 1: Detailed thromboelastography (TEG) tracings for the patient that suffered a pulmonary haemorrhage on day 10 of admission. All anticoagulation was subsequently stopped. CKH=citrated kaolin in heparinise, CFF=citrated functional fibrinogen. Normal reference ranges as provided by the TEG 6s operating manual.
